# Management of Ankle Fractures With Syndesmotic Disruption: A Survey of Orthopaedic Surgeons

**DOI:** 10.7759/cureus.16391

**Published:** 2021-07-14

**Authors:** Ryan G Rogero, Emmanuel M Illical, Daniel O Corr, Steven M Raikin, James C Krieg, Justin Tsai

**Affiliations:** 1 Orthopaedic Surgery, Lewis Katz School of Medicine at Temple University, Philadelphia, USA; 2 Orthopaedic Surgery, Rothman Orthopaedic Institute, Philadelphia, USA; 3 Orthopaedic Surgery, Einstein Healthcare Network, Philadelphia, USA; 4 Foot and Ankle Surgery, Rothman Orthopaedic Institute, Philadelphia, USA; 5 Orthopaedic Surgery, Rothman Orthopaedic Institute, New York, USA

**Keywords:** syndesmosis, ankle fracture, construct, protocol, demographics

## Abstract

Introduction: With no current “gold standard” fixation strategy for syndesmotic injuries and differences in preferred preoperative and intraoperative diagnostic techniques and criteria, methods of reduction, fixation constructs, and postoperative management, the goals of this study were to determine how orthopaedic surgeons currently manage ankle fractures with concomitant syndesmotic disruption, as well as to identify surgeon demographics predictive of syndesmotic management techniques.

Methods: This study was conducted as a web-based survey of foot and ankle fellowship-trained surgeons, Orthopaedic Trauma Association (OTA) members, and Canadian Orthopaedic Association (COA) members. The survey, sent and completed via the HIPAA-compliant Research Electronic Data Capture (REDCap) system, consisted of 18 questions: 6 surgeon demographic questions and 12 specific syndesmotic management questions regarding perioperative protocols and syndesmotic fixation construct techniques.

Results: One hundred and ten orthopaedic surgeons completed our survey. Years of practice and type of fellowship were found to be the variables that influenced perioperative syndesmotic management strategies the most, while a number of fractures operated on per year, country of practice, and practice setting also influenced management decisions. Additionally, 59% (65/110) surgeons indicated that the way they have managed syndesmotic injuries has changed at some point in their career, while 33% (36/110) specified that they could foresee themselves changing their management of these injuries in the future.

Conclusions: There was significant variability among responders in preoperative and intraoperative assessment technique, fixation construct, screw removal protocol, and postoperative weightbearing protocol. This study raises awareness of differences in and factors predictive of management strategies and should be used for further discussion when determining a potential gold standard for the management of these complex injuries.

## Introduction

Injuries of the tibiofibular syndesmosis accompany up to 10% to 37% of ankle fractures [1-3]. Symptomatic and unstable injuries are usually treated surgically, although identifying syndesmotic disruption and determining stability can often prove clinically challenging [3,4]. A national database analysis recently reported an increase in syndesmotic fixation with ankle fracture open reduction internal fixation (ORIF), suggesting a surge in surgeon recognition which leads to more operative treatment of these often subtle injuries [5].

Accurate reduction and proper fixation of these injuries are paramount, as malreduction results in significantly impaired functional outcomes, and a high correlation with the eventual development of post-traumatic arthritis [6,7]. Increasing emphasis in recent years has been placed on determining the optimal repair technique for unstable syndesmosis injuries, with good outcomes described using several different techniques [8,9]. Although the primary goal of treatment of ankle injuries with disruptions of the syndesmosis is to restore ankle joint alignment and stability [4,8], techniques currently vary in number, size, orientation, location, and types of devices implemented, as well as intraoperative and postoperative protocols [3,8].

With the increasing frequency of surgical treatment of syndesmotic injuries and no current consensus on management, it may be valuable for surgeons to understand patterns of usage of various techniques among orthopaedic surgeons. The purposes of this study are to determine how orthopaedic surgeons currently manage ankle fractures with concomitant syndesmotic disruption, as well as identify surgeon demographics predictive of syndesmotic management techniques. Our hypothesis is that some of the variations in treatment have to do with surgeon demographics, rather than patient- or injury-related factors.

## Materials and methods

An 18-question multiple-choice survey was developed (Tables 1 and 2) to assess how orthopaedic surgeons manage syndesmotic injuries. The first six questions pertained to surgeon demographic factors, including years of practice, country of practice, type of practice, fellowship(s) completed, surgical facility in which the majority of ankle fractures were performed, and self-reported number of ankle fractures operated on per year (Table 1). The next 12 questions asked surgeons to indicate their preferences regarding the pre- and intraoperative assessment of syndesmotic injury, construct choices, and postoperative protocols (Table 2). Several of the questions on the survey allowed for multiple answers.

**Table 1:** Orthopaedic Surgeon Demographics.

Question	Responses	N (%)
1. How many years have you been in practice as an attending orthopaedic surgeon?	0-5 years	38 (35)
	6-10 years	20 (18)
	11-15 years	13 (12)
	16-20 years	10 (9)
	>20 years	29 (26)
2. In which country do you currently practice?	United States	64 (58)
	Canada	41 (37)
	Other	5 (5)
3. What type of practice are you in?	Solo	10 (9)
	Private practice	16 (15)
	Private practice with academic opportunities	32 (29)
	Academic	44 (40)
	None of the above applicable	8 (7)
4. What fellowship(s) have you completed?	No fellowship	5 (5)
	Foot and ankle	27 (25)
	Trauma	45 (41)
	Sports	3 (3)
	Other	13 (12)
	Multiple fellowships	17 (15)
5. How many ankle fractures do you operate on per year?	Between 1 and 5	1 (1)
	Between 6 and 10	4 (4)
	Between 11 and 25	29 (26)
	Between 26 and 50	59 (54)
	50+	17 (15)
6. In what setting do you perform the majority of your ankle fracture ORIF?	Ambulatory surgery center, hospital-owned	5 (5)
	Ambulatory surgery center, privately owned without a personal financial stake	5 (5)
	Ambulatory surgery center, privately owned with a personal financial stake	9 (8)
	Hospital	91 (83)

**Table 2:** Syndesmotic Management Questions and Responses. Legend: Questions 7, 8, 10, 11, and 13 were able to have multiple answers selected. Questions 10-13 were optional based on surgeon's technique. The number (N) of surgeons responding to each optional question is listed.

Question	Responses	N (%)
7. For a Weber B ankle fracture where syndesmotic involvement is not explicitly evident, how do you assess the syndesmosis preoperatively?	Gravity stress test XR	32 (29)
	External rotation stress test XR	49 (45)
	Clinical examination	42 (38)
	Trial of weightbearing	28 (25)
	None of the above	16 (15)
8. How do you assess the syndesmosis integrity intraoperatively?	Direct visualization of the incisura	31 (28)
	Concomitant arthroscopic examination	6 (5)
	Cotton test	60 (55)
	External rotation stress test	85 (77)
	None of the above	2 (2)
9. For an ankle fracture with syndesmotic involvement, what is your typical construct in a patient without complicating factors?	Screws alone	64 (58)
	K-wire alone	1 (1)
	Suture button (TightRope) alone	27 (25)
	AITFL Internal Brace	0 (0)
	Combination of the above	18 (16)
10. If using screws, what construct for a non-complicated patient? (N=102)	3.5 mm	83 (81)
	4.0 mm	11 (11)
	4.5 mm	7 (7)
	Through plate	55 (54)
	1 screw	41 (40)
	2 screws	46 (45)
	3 cortices	34 (33)
	4 cortices	62 (61)
11. If using suture buttons (e.g., TightRope), what construct for a non-complicated patient? (N=70)	1	55 (79)
	2	15 (21)
	Through plate	35 (50)
12. If using screws, what is your practice regarding removal? (N=106)	Never remove	3 (3)
	No plan for removal unless symptomatic	53 (50)
	Remove at 3 months	20 (19)
	Remove at 4 months	27 (25)
	None of the above	3 (3)
13. If using both suture-buttons (e.g., TightRope) and screws, what are some factors that might lead you to use one or the other? (N=60)	The setting of surgery (e.g., hospital vs. ambulatory surgery center)	4 (7)
	Patient age	32 (53)
	Patient weight	30 (50)
	Patient with diabetes or other medical comorbidities relevant to bone health/healing	39 (65)
	The severity of fracture/injury	37 (62)
	Concomitant fracture	21 (35)
14. How long do you keep the patient non-weight bearing following syndesmotic fixation in a syndesmotic injury (either Maisonneuve or pure syndesmotic injury)?	Immediate weightbearing	3 (3)
	2 weeks	2 (2)
	4 weeks	4 (4)
	6 weeks	66 (60)
	8 weeks or greater	35 (32)
15. How long do you keep the patient non-weight bearing following syndesmotic fixation in a syndesmotic injury associated with lateral malleolar fractures?	Immediate weightbearing	4 (4)
	2 weeks	2 (2)
	4 weeks	2 (2)
	6 weeks	75 (68)
	8 weeks or greater	27 (25)
16. How long do you keep the patient non-weightbearing following syndesmotic fixation in a syndesmotic injury associated with lateral and medial malleolar fractures?	Immediate weightbearing	3 (3)
	2 weeks	2 (2)
	4 weeks	1 (1)
	6 weeks	68 (62)
	8 weeks or greater	36 (33)
17. Has the way you’ve managed syndesmotic injuries changed at some point in your career?	Too early in career to determine	24 (22)
	Yes	65 (59)
	No	21 (19)
18. Do you foresee yourself changing the way you manage syndesmotic injuries in the future?	Yes	36 (33)
	No	13 (12)
	Possible/unknown	61 (55)

The survey was transferred to the Research Electronic Data Capture (REDCap) system [10], and a public link to the survey was given to the Orthopaedic Trauma Association (OTA) and Canadian Orthopaedic Association (COA), as well as sent to email addresses of foot and ankle fellowship-trained surgeons. We considered this cohort of surgeons to be the most experienced in treating syndesmotic injuries, with an eye towards including a diverse population of orthopaedic surgeons.

Statistical analysis

All statistical analyses were performed using IBM SPSS Statistics for Windows, version 26 (IBM Corp., Armonk, NY, USA). Frequencies and percentages of surgeon demographics and responses were calculated and reported for each question. To determine surgeon demographics independently predictive of syndesmotic management techniques, binomial logistic regression analysis was used for questions with dichotomous answer selections, while multinomial regression analysis was performed for questions with more than two answer selections. The largest category for each surgeon demographic variable was used as the reference variable for each binomial logistic regression analysis, while the most popular answer was chosen as the reference category for multinomial analyses. Odds ratios (OR), 95% confidence intervals (95% CI), and p-values were reported only for significant findings of each regression analysis. Statistical significance was set at p ≤ 0.05.

## Results

One hundred and ten orthopaedic surgeons completed our survey. Demographic information of responding surgeons is listed in Table 1. Frequencies of syndesmotic management answers are detailed in Table 2. Significant findings for each question are summarized below, grouped into whether they pertain to preoperative, intraoperative, or postoperative management.

Preoperative assessment (question 7)

Question 7 asked surgeons about their preferred method of preoperative assessment of syndesmotic stability.

Surgeons with zero to five years of experience were more likely to utilize a gravity stress X-ray compared to surgeons with greater than 20 years of experience (0.106 [0.022, 0.522]; p=0.006). Surgeons based in the U.S. had a greater likelihood of using the external rotation stress X-ray compared to their Canadian counterparts (0.115 [0.025, 0.530]; p=0.006).

Surgeons with greater than 20 years of practice (4.471 [1.039, 19.242]; p=0.044) and surgeons in Canada (7.967 [1.741, 36.460]; p=0.007) both had increased odds of using a clinical examination for preoperative assessment compared to surgeons with zero to five years and surgeons in the U.S., respectively. Further, surgeons operating on 11 to 25 ankle fractures per year had decreased likelihood of using a clinical examination (0.155 [0.033, 0.736]; p=0.019) compared to surgeons operating on 26 to 50. Lastly, foot and ankle fellowship-trained surgeons were more likely to use a preoperative trial of weightbearing (7.550 [1.340, 42.534]; p=0.022) compared to trauma fellowship-trained surgeons.

Intraoperative assessment (question 8)

Question 8 asked surgeons regarding their preferred intraoperative assessment technique for determining syndesmotic integrity.

Surgeons with zero to five years of experience were more likely to select “direct visualization of the incisura” as a choice compared to those with greater than 20 years of practice (0.177 [0.033, 0.949]; p=0.043). Alternatively, foot and ankle fellowship-trained surgeons (18.368 [2.452, 137.607]; p=0.005) and surgeons operating on 11 to 25 fractures per year (5.326 [1.273, 22.285]; p=0.022) were both more likely to utilize direct visualization than trauma-trained surgeons and surgeons operating on 26 to 50 fractures, respectively.

Construct selection (questions 9-11, 13)

Question 9 asked participants to select their typical construct for syndesmotic fixation in a non-complicated patient with an unstable syndesmosis in the setting of an ankle fracture. No specification was given as to what a complicating factor would be.

Surgeons involved in solo practices (9.938 [1.041, 94.847]; p=0.046) or private practices with academic appointments (8.046 [1.697, 38.153]; p=0.009) were predictive of choosing suture-buttons (e.g., TightRope® [Arthrex Inc., Naples, FL]) over screws alone. Surgeons with 6 to 10 years of practice (8.419 [1.003, 70.652]; p=0.050) were more likely to use a combination of screws, suture-buttons, AITFL internal braces, or K-wires over screws alone.

Questions 10 asked responders to indicate their screw construct for syndesmosis fixation alone.

Surgeons with greater than 20 years of practice were less likely to use a 3.5 mm screw (0.077 [0.008, 0.723]; p=0.025) than those with zero to five years of experience, whereas foot and ankle fellowship-trained surgeons (33.428 [1.352, 826.463]; p=0.032) and those with multiple fellowships (44.146 [1.135, 1716.800]; p=0.043) were more likely to use 4.0 mm screws for ankle fractures with syndesmotic involvement than trauma-trained surgeons. Surgeons with zero to five years of experience and surgeons with a trauma fellowship were more likely to implement screws through a plate than surgeons with 16 to 20 years of practice (0.035 [0.003, 0.488]; p=0.013) and surgeons with multiple fellowships (0.076 [0.010, 0.548]; p=0.011), respectively. Furthermore, surgeons with greater than 20 years of practice were less likely to utilize four cortices (0.230 [0.057, 0.921]; p=0.038) than surgeons with zero to five years.

Question 11 asked about the construct of a suture-button device when used in an isolated syndesmotic injury.

Surgeons with a foot and ankle fellowship were more likely to use two suture-buttons (151.309 [2.994, 7645.946]; p=0.012), less likely to use one (0.011 [0.000, 0.536]; p=0.023), and more likely to place the suture-button through a plate (71.406 [1.409, 3618.212]; p=0.033) as opposed to trauma fellowship-trained surgeons. Surgeons in private practices with academic appointments were also less likely to use two suture-buttons (0.037 [0.001, 0.928]; p=0.045) compared to those in academic practices. Surgeons in purely private practice were more likely to use suture-buttons through a fracture plate (15.635 [1.080, 226.299]; p=0.044) compared to surgeons in academic practice. Finally, surgeons in Canada, when compared to those in the U.S. were less likely to use suture-buttons through a plate (0.023 [0.001, 0.786]; p=0.036), while those practicing 6 to 10 years were less likely to go through a plate (0.124 [0.017, 0.914]; p=0.041) compared to those practicing 0 to 5 years.

Question 13 asked responders to choose patient factors that would influence their decision on whether to use a suture-button device or screw.

Surgeons with foot and ankle fellowships were more likely to consider patient age when choosing a construct (28.310 [1.241, 645.688]; p=0.036) compared to trauma surgeons. Those practicing 11 to 15 years were more likely to consider patient weight (35.629 [1.099, 1155.150]; p=0.044) than those practicing zero to five years when choosing a construct. Canadian surgeons were less likely (0.029 [0.001, 0.715]; p=0.030) than surgeons in the U.S. to consider “diabetes or other medical comorbidities relevant to bone health/healing.”

Postoperative protocol (question 12, 14-16)

Question 12 polled participants regarding their postoperative management of a syndesmotic screw, if employed.

Half (50%) of responding surgeons reported having no plan for screw removal unless symptomatic, compared to 44% of surgeons saying they remove screws at three or four months postoperatively. Surgeons with a foot and ankle fellowship were more likely to select removal at four months or greater (11.719 [1.720, 79.863]; p=0.012) compared to having no plan for removal unless symptomatic.

Question 14, 15, and 16 asked surgeons to identify how long they would keep patients non-weightbearing for in-patients with pure syndesmosis injuries, and those with lateral malleolar and bimalleolar involvement, respectively. 

The majority of surgeons responded using six weeks of non-weightbearing for pure syndesmotic injuries (60%), lateral malleolar fractures with syndesmotic injuries (68%), and bimalleolar fractures with syndesmotic fixation (62%), while 5%, 6%, and 5% surgeons reported using immediate or only two weeks of non-weightbearing for these injuries, respectively.

Surgeons with six to 10 years of practice (11.225 [1.338, 94.148]; p=0.026) were more likely to keep patients weightbearing eight weeks or longer postoperatively compared to six weeks following pure syndesmotic injury. Similarly, surgeons in private practice (7.228 [1.007, 51.894]; p=0.049) were more likely to keep patients weightbearing eight weeks or longer compared to six weeks following bimalleolar ankle fractures with syndesmotic involvement.

Management trends (questions 17 and 18)

Questions 17 and 18 asked surgeons both whether their management of syndesmosis injuries have changed during their career and whether they would foresee or consider a change in the future. Fifty-nine percent of surgeons (65/110) responded “yes” to the former, and 33% (36/110) specified that they foresee themselves changing their management of these injuries in the future.

## Discussion

To the best of our knowledge, this study is the first to identify surgeon demographic factors that influence treatment patterns of syndesmotic injuries. Although it is well established that stable and anatomic syndesmotic fixation positively influences surgical outcomes [11,12], there still remains some debate among orthopedic surgeons regarding optimal treatment [13,14]. Variables in treatment strategies include a method of evaluating syndesmotic stability, fixation material, screw size, number of cortices engaged with screw, postoperative weightbearing protocol, and whether or not fixation is placed through a plate in pure syndesmotic injuries without associated fracture. A survey of trauma and orthopaedic surgeons in the Netherlands revealed a preference for 3.5 mm screws engaging three cortices, with routine removal at six to eight weeks in the majority of surgeons responding [14]. A similar study involving orthopaedic consultants in the United Kingdom demonstrated a preference for a screw as the fixation method, placed without compression [13].

Assessing syndesmotic stability can often prove difficult, obvious injuries notwithstanding. Methods described include external rotation stress tests (both manual and gravity), lateral stress test, intraoperative “Hook” test, and computed tomography [15-17]. One cadaveric study demonstrated that the lateral stress test produced a greater increase in tibiofibular clear spaced compared to an external rotation stress test [17]. Gravity stress tests have been shown to be as reliable as manual stress tests in detecting instability, which may obviate the need for increased radiation exposure to the examiner performing the latter [15]. Arthroscopic assessment of the syndesmosis has also shown to reliably detect instability, especially in the sagittal plane [18].

Perhaps the greatest variability in syndesmotic treatment patterns lies in the construct used. Traditionally, screws were used in a variety of different techniques. Several studies have demonstrated no biochemical advantage of one method over another - including the size of screw or cortices engaged [19,20]. Absorbable screws have been shown to be just as strong but demonstrated an increased incidence of foreign body reaction [21]. Using a screw introduces another longstanding debate: whether routine removal is warranted. A recent systematic review demonstrated no difference in clinical or radiographic outcome in patients who underwent screw removal [22]. There was a higher likelihood of recurrent diastasis with screw removal between six and eight weeks, and a higher likelihood of postoperative infection if preoperative antibiotics were not administered prior to the removal procedure. Manjoo et al. showed that while patients with an intact syndesmosis screw had worse functional scores compared to those with loose, fracture, or removed screws, there was no difference in functional scores between patients with removed screws and those with loose or fractured screws [23]. Based on their study, the authors concluded that screw removal was unlikely to provide benefit to patients with loose fractured screws.

More recently, suture-button type devices have been used as an alternative to rigid screw fixation. The theoretical benefit of these devices includes less potential for malreduction and retention of some physiologic movement at the tibiofibular joint [24-26]. Biomechanical studies have not demonstrated a clear advantage to using more than one suture button device [27], one brand of the device over the other [28], or using diverging directions when placing the device [8]. From a clinical perspective, two recent randomized controlled trials have demonstrated better functional scores in patients with suture button fixation compared to those with screw fixation [2,29]. A cost-effectiveness analysis of suture button versus syndesmotic screw fixation showed that a suture button was more cost-effective in the majority of cases [30]. Screw fixation was only more cost-effective if screws were removed less than 10% of the time, or if the cost of the suture button devices cost more than $2000.

Our survey reflected the diverse nature of syndesmotic injury management. There was significant variability among responders in preoperative and intraoperative assessment technique, fixation construct, screw removal protocol, and postoperative weightbearing protocol. Of all the surgeon demographic variables (Table 1), years of practice and fellowship type influenced the most amount of management variables. Those practicing longer were less likely to use a gravity stress exam prior to surgery and visualizing the incisura intraoperatively to assess the syndesmosis, compared to other methods. They were also more likely to avoid using smaller screws (3.5 mm), whereas those with fewer years of practice (0-10 years) were less likely to start weightbearing before eight weeks. Surprisingly, the country of practice or surgery setting had no significant influence on the decision to use screws or a suture button construct despite the perception that suture button devices are more expensive. Foot and ankle fellowship-trained surgeons were more likely to use suture-type devices through a plate, perhaps indicating a willingness to embrace newer technology.

Interestingly, a majority of surgeons indicated that their treatment strategies had changed within the last year and would possibly change in the future. Weaknesses in our study include a low number of responders and a skew toward responders with a trauma fellowship background as opposed to foot and ankle. There was also a disproportionate number of surgeons operating on ankle fractures predominantly in a hospital setting, limiting analysis of surgical settings as a factor in management decisions. Further research will hopefully lead to a greater consensus on a clinical efficacious and cost-effective treatment model for syndesmotic injuries.

## Conclusions

This survey study of certified orthopaedic surgeons demonstrated significant variability in preoperative, intraoperative, and postoperative approaches to the treatment of ankle fractures with syndesmotic disruption. Assessment technique, hardware choice, plan for hardware removal, and postoperative weightbearing protocol all varied among surgeon demographic groups. These differences in practice strategies and demographic factors associated with them can be used in further discussion and investigation of the most effective available treatment strategies.
